# Determining Infected Aortic Aneurysm Treatment Using Focused Detection of *Helicobacter cinaedi*

**DOI:** 10.3201/eid2807.212505

**Published:** 2022-07

**Authors:** Jien Saito, Emiko Rimbara, Shingo Inaguma, Chihiro Hasegawa, Shinji Kamiya, Akihiro Mizuno, Yoshiaki Sone, Tatsuhito Ogawa, Yukihide Numata, Satoru Takahashi, Miki Asano

**Affiliations:** Nagoya City University East Medical Center, Aichi, Japan (J. Saito, S. Inaguma, C. Hasegawa, S. Kamiya, A. Mizuno, Y. Sone, T. Ogawa, Y. Numata, M. Asano);; National Institute of Infectious Diseases, Tokyo, Japan (E. Rimbara); Nagoya City University, Aichi (S. Takahashi)

**Keywords:** Helicobacter cinaedi, bacteria, antimicrobial treatment, infections, aortic aneurysm, arteriosclerosis, Japan

## Abstract

We detected *Helicobacter cinaedi* in 4 of 10 patients with infected aortic aneurysms diagnosed using blood or tissue culture in Aichi, Japan, during September 2017–January 2021. Infected aortic aneurysms caused by *H. cinaedi* had a higher detection rate and better results after treatment than previously reported, without recurrent infection.

Infected aortic aneurysms account for 0.7%–3% of all aortic aneurysms and are associated with a 26%–44% mortality rate (*1*). No consensus exists about appropriate antimicrobial therapy and treatment duration for infected aneurysms. Moreover, determining surgical treatment in each case requires carefully considering the causative bacterium and the patient’s medical background (*2*). Recently, several cases of infected aortic aneurysms caused by *Helicobacter cinaedi*, a rare, difficult-to-detect causative bacterium have been reported (*3*).

First identified in 1984, *H. cinaedi*, a gram-negative rod with spiral morphology and bipolar flagella, is indigenous to the intestinal tract of humans and other animals (*4**,**5*). This bacterium produces a cytolethal distending toxin that invades epithelial cells (*6*) and is associated with bacteremia in compromised hosts and infected aortic aneurysms, mediated by bacterial translocation from the intestinal mucosa (*7**,**8*). Because of the high recurrence rate for *H. cinaedi* bacteremia, it is recommended that patients receive prolonged treatment of at least 3 months with appropriate antimicrobial drugs (*9*). We sought to determine the efficacy of treatment for infected aortic aneurysms through the focused detection of *H. cinaedi*.

## The Study

During September 2017–January 2021, we treated 10 patients with infected aortic aneurysms from a single center in Aichi, Japan. Diagnosis, including for recurrent aneurysms, was based on either positive culture or PCR of aortic tissue resected at the time of surgery or positive blood or puncture culture of an abscess caused by a hematogenous infection in patients who did not undergo open surgery and had clinical findings localized to the aortic aneurysm.

We started patients on antimicrobial therapy with meropenem when *H. cinaedi* was suspected or gram-negative rods were identified, then changed to sulbactam/ampicillin after confirming drug sensitivity. After 4 weeks of treatment or a negative inflammatory reaction, the antimicrobial treatment was switched to minocycline or amoxicillin/clavulanate for 3–6 months. If other causative bacteria were identified, antimicrobial drugs were changed based on drug sensitivity results and continued for 3–6 months. Rifampin-soaked graft replacement was the first-choice surgical treatment, irrespective of causative bacterium.

Among the 10 patients with infected aortic aneurysms, *H. cinaedi* was the causative bacterium in 4, *Staphylococcus aureus* in 3, *Salmonella enterica* serovar Enteritidis in 1, and *Enterobacter cloacae* in 1. Treatment included vascular replacement in 7 patients (2 with *H. cinaedi*), endovascular stent grafting in 1 (with *H. cinaedi*), and medical treatment in 2 patients (1 with *H. cinaedi*). One patient with aortic rupture and *Salmonella* Enteritidis infection died postoperatively from multiorgan failure; the other 9 patients had good courses of recovery without recurrence (Table 1).

**Table 1:** Demographic and clinical variables of 10 patients with infected aortic aneurysms with or without *Helicobacter cinaedi*, Aichi, Japan, September 2017–January 2021*

Variables	Total, n = 10	*H. cinaedi*, n = 4	Non–*H. cinaedi*, n = 6
Age, median (IQR)	77 (70.5–83.0)	77.5 (70.0–84.3)	77 (71.0–82.3)
Sex			
M	8	3	5
F	2	1	1
Comorbidity			
Diabetes mellitus	3	2	1
Chronic kidney disease	1	1	0
Cancer	2	1	1
Steroid use	1	0	1
Sign or symptom			
Pain	8	3	5
Fever	7	3	4
Shock	1	0	1
Laboratory findings, median (IQR)			
Leukocytes, × 10^3^ cells/μL	9.6 (8.8–11.0)	8.4 (7.4–9.5)	1.1 (9.5–17.2)
C-reactive protein, mg/dL	7.6 (5.1–22.1)	4.3 (3.8–5.1)	21.6 (11.0–23.6)
Procalcitonin, ng/dL	0.19 (0.05–0.76)	0.14 (0.04–0.38)	0.38 (0.08–1.53)
Aneurysm diameter, mm, median (IQR)	40.5 (32.8–44.8)	32.5 (25.5–39.8)	44.5 (37.3–55.5)
Aneurysm location			
Thoracic aorta	1	1	0
Thoracoabdominal aorta	1	0	1
Abdominal aorta	8	3	5
Aneurysm form			
Saccular	7	4	3
Fusiform	3	0	3
Rupture	3	0	3
Aortoduodenal fistula	2	0	2
Surgery			
Emergency	3	0	3
Urgent	2	1	1
Elective	5	3	2
Endovascular	1	1	0
Nonsurgical treatment only	2	1	1
Death	1	0	1

*IQR, interquartile range.

**Figure 1:**
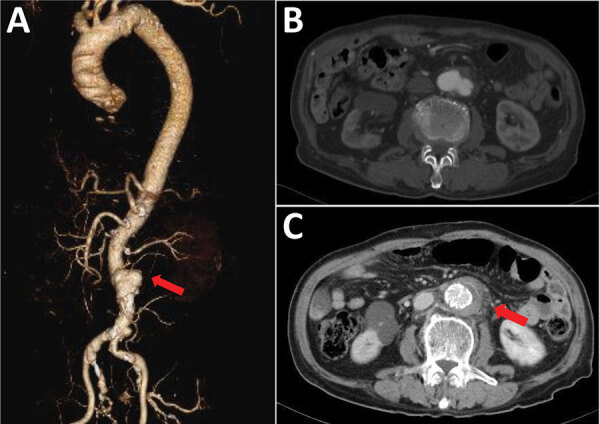
Contrast-enhanced computed tomography imaging for Case-patient 3 in the *Helicobacter cinaedi* group of 10 patients with infected aortic aneurysms with or without *H. cinaedi*, Aichi, Japan, September 2017–January 2021. A, B) The infrarenal aortic aneurysm had a maximum short diameter of 39 mm and a cystic protrusion of 19 mm (arrow in panel A) before the operation. C) After the operation, the adipose tissue concentration increased around the aneurysm (arrow).

All 4 of the *H. cinaedi*–infected case-patients were immunocompromised (diabetes mellitus, chronic kidney disease, cancer). However, their clinical findings were mild, and C-reactive protein levels tended to be low (*H. cinaedi*/non–*H. cinaedi* median 4.3/21.6 mg/dL) at hospital admission. All patients had saccular aneurysms and severely calcified aortas. Surgical findings showed highly adherent areas around the aneurysms with intimal defects but without abscess formation. Pathological examination revealed severe lymphocytic infiltration in the aneurysmal wall with loss of elastic fibers. In contrast, 3 of 5 patients in the non–*H. cinaedi* group showed abscesses and hematomas around the infections (Figure 1).

**Figure 2:**
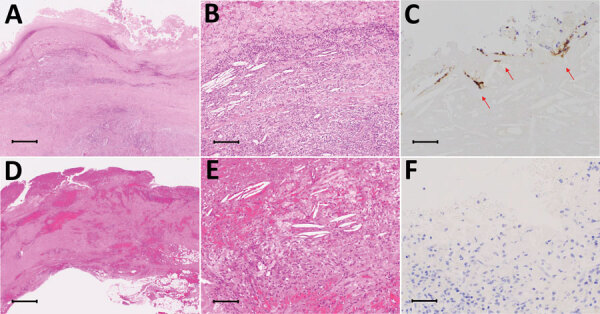
Comparison of images from patients in the *Helicobacter cinaedi* group with patients from the non–*H. cinaedi* group among 10 patients with infected aortic aneurysms with or without *H. cinaedi*, Aichi, Japan, September 2017–January 2021. Immunohistochemistry was performed on the whole cell lysates of *H. cinaedi* strain MRY08-1234 isolated from immunocompromised patients in Japan by raising anti–rabbit *H. cinaedi* IgG. One of 2 case-patients with resected tissue in the *H. cinaedi* group had positive immunostaining (patient 1). A–C) Case-patient 1 in the *H. cinaedi* group. D–F) Case-patient 4 in the non–*H. cinaedi* group. In images from both patients, lymphocyte and neutrophil infiltrates, cholesterol clefts, foam cells, plasma cells, foreign body giant cells, and hemosiderin deposition are visible (A, B, D, E; hematoxylin & eosin). Immunohistochemistry stain shows of *H. cinaedi* organisms in the aortic intima (arrow in C) and negative results (F). Scale bars: 1,000 µm in A, D; 100 µm in B, E; 50 µm in C, F.

We performed blood cultures using the BacT/Alert system (bioMérieux, https://www.biomerieux.com) and grew microaerobic cultures in the presence of hydrogen using a commercial hydrogen generator (SUGIYAMA-GEN Co., Ltd., http://sugiyama-gen.com). In the *H. cinaedi* group, we used multilocus sequence typing (MLST) to identify the subtype, and we immunostained aortic tissues with antiserum against the whole-cell lysate of *H. cinaedi* raised in rabbits (Figure 2; Appendix Figure) (*10*).

Among the *H. cinaedi* patients, we were able to subculture isolates from 3; the range of blood culture growth times, 75.3–160.8 h (median 90.6 h), was longer than that among the non–*H. cinaedi* patients, 12.3–28.3 h (median 12.3 h). Drug susceptibility testing demonstrated levofloxacin-resistant *H. cinaedi*, although the sequence type on MLST was different in each case (Tables 1, 2).

**Table 2:** Bacteriological examination, method of treatment, and outcomes for 10 patients with infected aortic aneurysms with or without *Helicobacter cinaedi*, Aichi, Japan, September 2017–January 2021*

Patient no.	Age, y/sex	Location	Blood cultures	ID	Comorbidity	Procedure‡	Antimicrobial use period	Outcome
*H. cinaedi* group, n = 4†								
1	72/M	Aortic arch	75.3/negative	ST6	DM	TAR (positive)	6 mo	Survival at 4 y
2	64/F	Right common iliac	Negative	Unknown	DM	Y graft (negative)	6 mo	Survival at 3 y
3	83/M	Infrarenal aorta	86.5/160.8	ST18	Malignant lymphoma	EVAR (NA)	6 mo	Survival at 2 y
4	88/M	Infrarenal aorta	90.6/93.0	ST21	CKD	Medical treatment only (NA)	3 mo	Survival at 1 y
Non–*H. cinaedi* group, n = 6§								
1	70/M	Infrarenal aorta	Negative	MSSA	None	Y graft	6 mo	Survival at 4 y
2	74/M	Infrarenal aorta	Negative	*Listeria monocytogenes*	None	Y graft	6 mo	Survival at 4 y
3	67/M	Infrarenal aorta	12.6/12.6	*Enterobacter cloacae*	AEF	Y graft	6 mo	Survival at 3 y
4	83/M	Infrarenal aorta	12.3/12.3	MSSA	DM	Y graft	3 mo	Survival at 3 y
5	86/F	Thoracoabdominal aorta	Negative	MSSA	None	Medical treatment only	3 mo	Survival at 1 y
6	80/M	Infrarenal aorta	28.3/28.3	*Salmonella enteritidis*	None	Y graft	Until death	Death at POD 5

*DM, diabetes mellitus; CKD, chronic kidney disease; TAR, total arch replacement; EVAR, endovascular aortic repair; NA, not applicable; MSSA, methicillin-susceptible *Staphylococcus aureus*; AEF, aorto-enteric fistula; POD, postoperative day; ST, sequence type. †16S RNA results were positive for all patients. ‡Y graft replacement for abdominal aortic aneurysm. §Tissue culture results for all patients returned in 1 d; patient 5 had an abscess.

## Conclusions

Infected aortic aneurysms caused by *H. cinaedi* are increasingly being recognized, especially in Japan, although the detection rate remains low (*3*). One study reported 734 cases of infected aortic aneurysms caused by various organisms, including *Salmonella*, *Staphylococcus*, *Streptococcus*, and *Escherichia coli*, but not *H. cinaedi* (*1*). Infected aortic aneurysms are a critical disease with high mortality, and whereas identifying the causative bacteria is effective in determining treatment, 23.3%–25% of cases are caused by unidentified bacteria (*1*). In our study, the detection rate for *H. cinaedi* in infected aortic aneurysms was high, 40%, although the absolute number of cases was small. Of note, infections caused by *H. cinaedi* all showed good clinical courses. *H. cinaedi* is known to cause nosocomial infections; however, nosocomial infections were ruled out as mode of infection here because the 3 isolates that underwent MLST had different sequence types (*11*).

The Bactec FX system (https://www.bd.com), widely used for blood culturing of *H. cinaedi*, is generally considered to be more sensitive than the bioMérieux BacT/Alert system (*8*). Nevertheless, by assuming *H. cinaedi* was the causative bacterium of the infected aortic aneurysms for our patients and simply allowing a longer incubation period of 10 days versus the usual 5 days, the BacT/Alert system detected the bacterium in 3/4 cases, comparable to the Bactec FX system (*12*). It is sometimes difficult to grow bacterial subcultures in microaerophilic conditions; however, adding 5%–10% hydrogen effectively helps form characteristic thin-spread colonies (*4*). PCR reliably detects and identifies species, but matrix-assisted laser desorption/ionization time-of-flight mass spectrometry is also useful (*13*), although unfortunately this testing method results in a time lag between initiating treatment on and identifying bacteria.

*H. cinaedi* infects atherosclerotic sites, leading to the progression of atherosclerosis through lipid accumulation (*5*,*14*). Progression is slow, often taking months, and leads to no clinical findings even if a local infection is established (*7*,*15*). Considering the slow, localized progression and difficulty of detection of atherosclerosis associated with *H. cinaedi* infection, aortic aneurysms thought to be noninfectious might actually be infected by *H. cinaedi*. Clarifying the relationship between this bacterium and atherosclerotic diseases might lead to additional treatments.

No infected aortic aneurysms caused by *H. cinaedi* have resulted in rupture, and cases usually pass without recurrence following an appropriate period of antibiotic treatment. Because nonsurgical treatment has been shown to be effective, open surgery during the acute phase of infection might be overindicated. Endovascular stent grafting, a less invasive treatment than the standard vascular replacement procedure, followed by use of appropriate antimicrobial agents might successfully complete treatment (*3*). This treatment is beneficial among aging patients, and it is hoped that the presence of these bacteria in infected aortic aneurysms will be widely recognized, with treatment and diagnosis proceeding simultaneously.

In summary, although *H. cinaedi* is a relatively rare cause of infected aortic aneurysms, it might be overlooked because of its low initial detection rate. Detection during treatment initiation can improve patient life expectancy by enabling effective antimicrobial therapy and expanding treatment options.

Appendix Additional information about determining treatment for infected aortic aneurysms through the focused detection of *H. cinaedi*.
