# Supplementary material for: Determining Infected Aortic Aneurysm Treatment Using Focused Detection of Helicobacter cinaedi
Source: Emerg Infect Dis. 2022 Jul;28(7):1494–8. doi: 10.3201/eid2807.212505 (PMC9239880; doi:10.3201/eid2807.212505)
Supplement: Appendix — Additional information about determining treatment for infected aortic aneurysms through the focused detection of H. cinaedi. [file 21-2505-Techapp-s1.pdf]

Article DOI: https://doi.org/10.3201/eid2807.212505

Determining Infected Aortic Aneurysm Treatment Using Focused Detection of *Helicobacter cinaedi*

Appendix

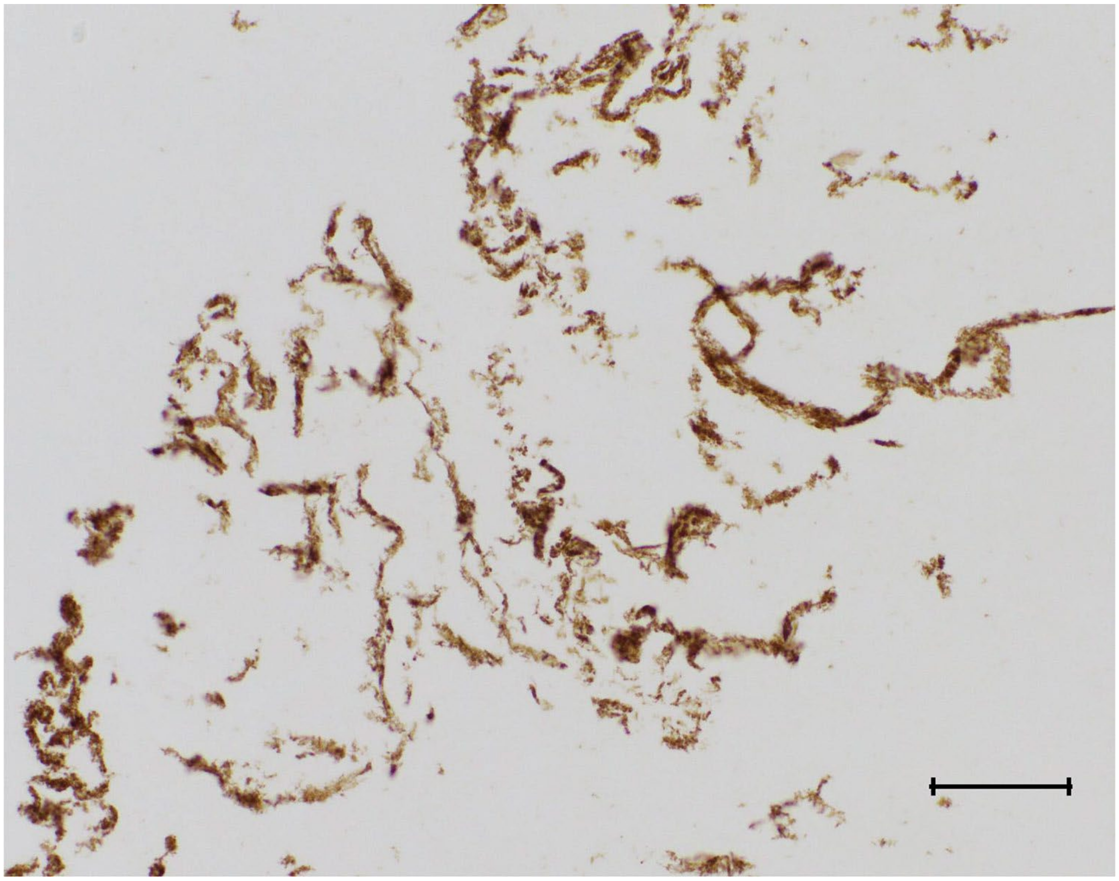

Appendix Figure. Positive control of immunohistochemistry; *Helicobacter cinaedi* clumps (MRY08-1234). Scale bar: 50 µm.
